# Bilingualism Does Not Hinder Grammatical Development in Down Syndrome: Evidence from a Sentence Repetition Task

**DOI:** 10.3390/bs15060791

**Published:** 2025-06-09

**Authors:** Alexandra Perovic, Katie Levy, Inès Aertsen, Andrea Baldacchino

**Affiliations:** 1 Division of Psychology and Language Sciences, University College London, London WC1E 6BT, UK; andrea.baldacchino.18@ucl.ac.uk; 2 Lewisham and Greenwich NHS Trust, London SE6 4JF, UK; katie.levy@nhs.net; 3 Gesondheetszentrum Greiweldeng, 5427 Greiveldange, Luxembourg; ines.aertsen@gezeg.lu

**Keywords:** Down syndrome, bilingualism, expressive grammar, sentence repetition

## Abstract

Despite the growing number of bilinguals worldwide, research on how bilingualism influences grammatical development in children with learning disabilities remains limited. This may be due to challenges in assessing language in these children, given the heterogeneity of their disabilities, lack of appropriate tools, and variability in language background and exposure common in bilingual populations. This pilot study investigates grammatical abilities in bilingual versus monolingual children with Down syndrome using the LITMUS Sentence Repetition Task, specifically designed for bilingual populations. Sentence repetition tasks are widely used for assessing grammar in neurotypical children and children with language impairments and are part of many omnibus language assessments. Ten children with Down syndrome aged 5–8 were recruited: five bilingual, speakers of British English and various home languages, and five monolingual, age- and language-matched. Both groups produced a high proportion of ungrammatical repetitions, with more omissions of verbs than nouns, function words than content words, and significant difficulties producing complex structures such as relative clauses, wh-questions, and passives. However, qualitative analyses showed that bilingual children speaking morphologically rich home languages (e.g., Polish, Greek) appeared to have fewer difficulties with some function words (e.g., prepositions) and were able to produce complex structures like passives and wh-questions, unlike their monolingual peers. Although the small sample limits generalisability, two insights emerge: First, sentence repetition may be of limited use in assessing expressive grammar in children with Down syndrome due to frequent ungrammatical responses. Second, while both groups showed similar challenges, bilingualism—especially with richly inflected home languages—may support specific grammatical skills. These findings support existing evidence that bilingualism does not hinder grammatical development in children with Down syndrome and suggest that parents should not avoid dual-language input. Further research is needed to determine whether bilingualism confers specific benefits in grammatical morpheme use and complex syntactic constructions.

## 1. Introduction

Down syndrome (DS), most often caused by triplication of chromosome 21, is one of the most common genetic causes of learning disability. Although language abilities in individuals with DS vary widely, they are typically very delayed and generally poorer than would be expected based on non-verbal mental age (NVMA) (e.g., Perovic, 2006; Næss et al., 2011). Grammar appears to be a particular area of weakness. Studies of spontaneous speech have revealed significant difficulties with the production of grammatical morphology (e.g., Eadie et al., 2002; Neitzel, 2024): English-speaking individuals with DS often omit inflectional morphemes (e.g., *-s*, *-ed*) and function words (e.g., auxiliary *is*, *was*), which are crucial for marking tense and agreement, leading to ungrammatical utterances such as “John run” or “Mary writing” (Laws & Bishop, 2004). Comprehension and production of complex syntactic structures have also been reported as exceptionally challenging, though they are less commonly studied in DS. Relying on different experimental methods, Ring and Clahsen (2005), Joffe and Varlokosta (2007), and Perovic and Wexler (2019), among others, report extensive difficulties in the comprehension and production of passive constructions and wh-questions in both children and adults. These constructions involve syntactic movement, which results in changes to word order and, in some cases, morphological modifications. In passive constructions, the object of the verb in the active sentence is moved to the subject position, and the auxiliary *be* marked for the relevant tense is inserted, as in active: “*Peter followed John*” → passive: “*John was followed by Peter*”. In object wh-questions, the object is replaced by a wh-word and moved to the front of the sentence, requiring auxiliary inversion, e.g., the declarative “*Peter is following John*” becomes “*Who is Peter following?*” If no auxiliary is present, as in the declarative “*Peter follows John*”, a dummy auxiliary *do* marked for tense is inserted, “*Who does Peter follow?*”. Both passives and wh-questions are known to be challenging for young typically developing (TD) children as well as individuals with developmental disorders, though different patterns of difficulty emerge related to the syntactic mechanisms involved in each construction (see Guasti, 2017 for details). English-speaking TD children may struggle with object wh-questions (“*Who is Peter following?*”) until at least the age of 5, though they find subject wh-questions easier (“*Who is following Peter?*”). With regards to passives, 4-year-old TD children show relatively strong performance with passives involving actional verbs (e.g., “*The children were taken to the office*”) but find passives of psychological verbs difficult at least until the age of 8 (e.g., “*She was seen in the morning*”). Similar patterns of difficulty with distinct types of passives and wh-questions have been found for children with delayed or impaired language, e.g., developmental language disorder (Guasti, 2017); thus, it is unsurprising that these constructions have been reported to be difficult for monolinguals with DS.

The question often posed in the clinical literature is whether bilingualism^1^ slows down an already slow process of language development (e.g., Uljarević et al., 2016). Typically developing bilinguals may have smaller vocabularies in each individual language compared with their monolingual peers, given that dual-language input may result in less exposure per language. However, when the vocabularies of both languages are combined, the total vocabulary size is often comparable with, or even larger than, that of monolinguals (see Byers-Heinlein et al., 2024, for review). An equally valid question is whether bilingualism can have an advantageous effect on language development. In typical development, there is now a wealth of literature arguing for the bilingualism advantage for a number of cognitive and language processes (e.g., Bialystok, 2001; Kovács, 2009). Crucially, it has been argued that metalinguistic skills of young bilinguals may be *enhanced* compared with monolinguals (although, see the recent literature questioning the strength of bilingualism advantage claims, e.g., Lehtonen et al., 2023). For neurotypical children, bilingual advantage has been claimed in the domains of morphological skills (Barac & Bialystok, 2012; Papastefanou et al., 2019) as well as for children with a language disability, such as dyslexia (Vender et al., 2018). If it is the case that bilingualism may facilitate the acquisition of morphosyntax, one of the most challenging areas for children with DS, then research into the effects of bilingualism on language development in DS is critical.

Despite its obvious research and clinical importance, the topic of bilingualism in DS remains severely under-researched. The few existing studies have primarily relied on standardised tests or natural language samples. While informative, these methods offer limited insight into children’s syntactic competence and often fail to capture their ability to produce or comprehend complex grammatical structures. Moreover, these studies often include participants of a wide age range, from early childhood to adolescence, which complicates the interpretation of results given the substantial language delays associated with DS. Importantly, most existing research has focused on individuals growing up in structured bilingual contexts where language exposure is supported by official government and education policies, such as English and Welsh in Wales, UK, or English and French in Canada.

In the following sections, we provide an overview of studies investigating language abilities in bilingual children with DS, identifying gaps in the literature, before presenting the rationale for the current study.

### 1.1. Grammar in Bilinguals with DS

In one of the first studies on the topic of bilingual language development in DS, Kay-Raining Bird et al. (2005) compared language abilities in eight English–French bilinguals with DS, aged 4; 7–11; 5 and 14 monolingual English speakers with DS, aged 2; 7–8; 5 (in addition to young typical controls, aged 2–3; 9). The two groups with DS did not differ significantly on standardised tests of English receptive vocabulary and receptive and expressive grammar, nor on expressive vocabulary. Importantly, no differences were revealed on measures derived from language sampling: mean length of utterance (MLU), the total number of words, or the number of different words. Feltmate and Kay-Raining Bird (2008) looked more closely at four bilinguals with DS from the same sample, placing them in triads with monolinguals with DS and TD bilinguals, matched on NVMA and exposure to the societal language. No difference between monolingual and bilingual children with DS were observed; the groups were heterogeneous in terms of MLU, but both groups were consistently poorer on measures of morphosyntax than typical controls. While no difference was noted in the number of verbs produced by monolinguals and bilinguals with DS, the monolinguals in this study displayed greater verb diversity than the bilinguals.

In a related study by the same research group, children and adolescents with DS were compared with NVMA-matched typical bilingual and monolingual controls on syntactic bootstrapping tasks involving unfamiliar nouns and verbs (Cleave et al., 2014). The age range of participants with DS was even wider, extending from 5 to 14 years in monolinguals (n = 12, mean age 10; 10) and from 5 to 19 years in bilinguals (n = 14, mean age 12; 5), compared with typical controls whose mean age was 4; 1. No significant differences were found between bilingual and monolingual participants in either the DS or TD groups; however, both children and adolescents with DS performed better on nouns than on verbs.

Two recent studies in Wales compared the language abilities of Welsh–English bilingual children with DS. Ward and Sanoudaki (2021) assessed ten bilingual and ten monolingual children with DS (aged 5; 5–13; 9), along with younger neurotypical controls, using a standardised omnibus language test (CELF-P-2: Wiig et al., 2004). No significant differences were found between bilingual and monolingual groups in verbal or non-verbal abilities, although the use of a broad measure limited insights into participants’ detailed grammatical profiles. In a follow-up study, Ward and Sanoudaki (2023) included a larger sample of 25 bilingual children with DS (aged 5; 5–16; 9) and examined whether the degree and age of exposure to Welsh predicted English language proficiency. Controlling for non-verbal cognition, short-term memory, and socioeconomic status, they found no significant effect of Welsh exposure on English receptive or expressive skills.

Finally, in the only study where English was not the societal but a home language, Katsarou and Andreou (2019) compared eight Greek–English bilinguals with DS, aged 4–8, with eight Greek-speaking monolingual peers, on a standardised task assessing articulation, vocabulary, and morphosyntactic comprehension in their societal language, Greek. However, no details were provided about the morphosyntax component of the task, nor any patterns of difficulty in the participants, making it unclear which grammatical structures were tested and what difficulties were observed. Again, no significant differences were found between groups.

The findings reviewed here indicate that bilingual children with DS acquire societal language at the same rate as their monolingual peers, suggesting that bilingualism is not detrimental to their language development. However, the bilingual contexts studied, English–French in Canada, English–Welsh in Wales, or Greek–English in Greece (where the minority language, English, is likely perceived as prestigious), are far from representative of the communities where children’s home language is unsupported by the educational and government systems and may be viewed less prestigious than the societal language. In urban environments such as London, UK, where hundreds of languages are spoken, families may face pressure to prioritise the dominant language for access to services (despite clinicians encouraging home language maintenance; see Sharpe & Perovic, 2024), while home languages receive less institutional support. Furthermore, the age range of the children with DS included in the above studies is typically too wide, covering early childhood (e.g., 4; 7) to adolescence (e.g., 19). This variation masks the effects of language delays common in individuals with DS, making the interpretation of results difficult. Finally, previous methodologies do not allow for fine-detailed analyses of grammatical knowledge, thus limiting the possibility of making accurate comparisons that may highlight specific differences between bilinguals and monolinguals. Standardised omnibus assessments are too broad to identify specific grammatical challenges faced by bilingual children with language impairment (see Schaeffer et al., *in press*). Similarly, while spontaneous language samples provide valuable insights, again they fail to capture competence in specific grammatical domains: children naturally avoid challenging structures, making it difficult to determine whether the absence of complex syntactic structures like passives or wh-questions indicates the actual lack of mastery of such structures.

### 1.2. The Current Study

Given the gaps in the literature, this study examines expressive grammar in a sample of bilingual children with DS aged 5; 9–8; 3, growing up in London, a multicultural environment where English is the dominant language in educational and clinical settings. Their grammatical skills are compared with those of monolingual peers with DS, closely matched in age, receptive vocabulary, receptive grammar, non-verbal abilities, and current speech and language therapy intervention. By narrowing the age range and controlling for cognitive and language abilities, we aim to minimise the effects of developmental delay and individual variability typical of DS.

To explore potential bilingualism-related effects on grammar, we use the LITMUS Sentence Repetition (SRep) Task (Marinis & Armon-Lotem, 2015), designed specifically for multilingual children, and adapted to the linguistic properties of the target language. This is the first study to apply the LITMUS SRep to children with DS; while sentence repetition is a common subtest of standardised omnibus tests administered to children with DS (e.g., CELF), unlike generic repetition tasks, LITMS SRep targets structures known to be challenging for children with language impairment, such as different types of passives and wh-questions, which are rarely produced in spontaneous speech. The task provides rich quantitative and qualitative data on grammatical morphology, as well as complex syntax, the latter of which has not been systematically investigated in bilinguals with DS.

This paper addresses the following research questions: How do the expressive grammatical skills of bilingual children with DS compare to those of monolingual children with DS, as assessed by a sentence repetition task? What insights does this method provide into the impact of bilingualism on grammatical morphology and mastery of complex syntactic structures in this population?

## 2. Materials and Methods

### 2.1. Participants

Participants, all with a confirmed diagnosis of standard trisomy 21, were recruited from a non-profit organisation that provide weekly speech and language therapy and occupational therapy sessions for children with DS in London, England: five monolingual English-speaking children DS, aged 5; 4–8; 9 (M = 6.78, SD = 1.27), and five bilingual children with DS aged 5; 9–8; 3 (M = 6.7, SD= 1.08), who spoke English and another language, Greek (B1), Somali (B2), Swedish (B3), Indonesian (B4), and Polish (B5), exposed to both languages from birth (see Table 1 for details). The children were identified as monolingual or bilingual by the members of staff who knew the families well. No significant difference in age between the groups was observed (t(8) = −0.11, *p* = 0.91). All participants were male, except for one monolingual female (M4).

### 2.2. Materials

Our battery consisted of a sentence repetition task and three standardised measures of general verbal and non-verbal abilities administered to all participants and a bilingualism background questionnaire for parents of bilingual participants.

Table 1 shows participants’ ages and raw and standard scores on the Matrices subtest of the Kaufman Brief Intelligence Test (K-BIT; Kaufman & Kaufman, 1990) measuring non-verbal intelligence, the British Picture Vocabulary Scale 3 (BPVS3; Dunn et al., 2009), which assesses receptive vocabulary, and the Test for Reception of Grammar 2 (TROG-2; Bishop, 2003), assessing receptive grammar.

An independent samples *t*-test revealed no significant difference on raw scores on the measures assessing non-verbal intelligence (KBIT Matrices) (t(8) = 0.15, *p* = 0.88) (bilinguals: M = 12.2, SD = 3.11; monolinguals: M = 11.8, SD = 5.26), receptive vocabulary (BPVS3) (t(8) = −1.859, *p* = 0.1) (bilinguals: M = 46.2, SD = 17.85; monolinguals: M = 65.4, SD = 14.66), or receptive syntax (TROG-2) (t(8) = −1.64, *p* = 0.14) (bilinguals: M = 1.8, SD = 2.05; monolinguals: M = 3.8, SD = 1.79).

Interestingly, the mean non-verbal IQ and standard scores on vocabulary and grammar for both groups appeared to be at the higher end for the population with DS. A likely explanation is that the children were all recipients of regular speech and language therapy from birth, in a highly stimulating setting. Many of the children had a history of hearing loss, typical for individuals with DS (Roizen, 2007). The following information was obtained from the professionals working with the children: B1 and B4 both had moderate hearing loss and wore hearing aids (HAs) during assessment; B2, B3, and M1 had hearing within the typical range; B5 had moderate hearing loss at the time of assessment and had bilateral HAs but did not wear them; M2 wore bilateral HAs; and M3, M4, and M5 all had previous hearing loss but did not wear HAs.

To gather information about the bilingual children’s language history and exposure, parents were asked to complete the Questionnaire for Parents of Bilingual Children (PaBiQ Tuller, 2015). However, only three parents completed the questionnaire in full, and a fourth parent completed it partially; therefore, this analysis is not included here. The following information was obtained via the available questionnaires and from the members of staff of the service: all participants were exposed to both English and a home language from birth; for two participants whose questionnaires were provided, the mother but not the father spoke the home language in addition to English; for the third participant whose questionnaire was provided, both parents spoke the home language and English; and for the two bilingual participants whose parents did not complete or only partially completed the PaBiQ, their exposure to the home language came from their fathers, while their mothers were English monolinguals.

The experimental protocol used a sentence repetition task that was developed as part of COST Action IS0804, the Language Impairment Testing in Multilingual Settings (LITMUS). The LITMUS-SRep Task (Marinis & Armon-Lotem, 2015) was designed to include language-independent syntactically complex structures (e.g., embedding and/or syntactic movement) known to be problematic for children with language impairment cross-linguistically (e.g., wh-object questions and object relative clauses), language-specific structures known to be difficult for children with language impairment in each language (e.g., passives, auxiliaries, and modals, in English), and subject–verb–object (SVO) structures as control sentences. Consisting of 30 items, the instrument controls for sentence length and vocabulary to ensure consistency within and across languages (see Table 2 for examples of each sentence structure).

### 2.3. Procedure

Participants were assessed in a quiet room at the premises of the centre, where children came for their once-weekly therapy classes, over the course of two sessions. Tests were administered in the following order: BPVS3, KBIT, TROG2, and LITMUS SRep. The tests were administered, scored, transcribed (for SRep), and checked for interrater reliability by two postgraduate students, one linguist, and one qualified speech and language therapist. Following the advice of clinicians who worked with the children weekly, we decided not to follow Marinis and Armon-Lotem’s (2015) protocol of presenting stimuli on a laptop. Instead, researchers read the stimuli aloud to participants, as this was considered the best way to maintain their attention. Responses were audio-recorded and not video-recorded, again to maintain participants’ attention.

### 2.4. Scoring

Following Marinis and Armon-Lotem (2015), participants’ utterances elicited by the LITMUS SRep Task were scored using a 0–3 scale (maximum score: 90). A score of 3 was given for verbatim repetition, 2 for one change, 1 for two or three changes, and 0 for more than three changes. Phonological errors were ignored, as per the scoring guidelines.

However, the 0–3 scoring method proved unfeasible due to the extremely high number of errors—seven out of ten participants scored only 3 or lower out of 90. To avoid a floor effect, we instead counted the total number of errors for each participant, which were used in the statistical analyses outlined below.

Following Polišenská et al. (2015), we also counted the total numbers of content words (CWs) and function words (FWs) repeated correctly and took note of any omissions, substitutions, and additions.^2^ These were then converted into percentages, dividing the numbers by the total number of target words. If a CW or FW was missing from the utterance, it was counted as an omission; if it was present, regardless of the word order, it was included in the count as repeated. If a word was produced with an incorrect inflection, for instance the verb ‘kick’ in “*Cow kick donkey*” for target “*The cow was kicked by the donkey*”, the verb form was still included in the CW count.

Substitutions were included within both CWs and FWs; e.g., in “*The boy must clean floor kitchen*” for the target “*The boy must sweep the floor in the kitchen*”, “*clean*” was included in the CW count.

Within CW and FW categories, correct repetitions/errors were broken down into syntactic categories/grammatical morphemes to allow for the identification of patterns across groups. Obligatory contexts were considered when calculating FWs and morphemes produced or omitted. For free morphemes, if the word or phrase modified by the participants had been omitted, this was not counted as an obligatory context and was therefore not scored as an omission. For instance, for the utterance “*bananas in the park*” produced instead of the target “*They are eating the bananas in the park*”, the auxiliary “are” was unnecessary, because the verb was absent. However, if the response was “*eating bananas in the park*”, here the auxiliary is omitted in an obligatory context. Likewise, for bound morphemes, if the target word to which they were attached had been omitted, this was not counted as an obligatory context; e.g., in “*The children sweets they tasted*”, for the target “*The children enjoyed the sweets that they tasted*”, there was only one obligatory context for -ed (*tasted*) because the first verb was omitted.

Given our small sample size, traditional statistical analyses may obscure clinically meaningful variation in this population. Because group analyses are likely insufficient, we also qualitatively examine individual performance profiles, particularly valuable when studying diverse language abilities typical of individuals with DS.

## 3. Results

While the completion rate was high, with participants providing no response to only 12 of the 300 total sentences, both groups produced an exceptionally high proportion of incomplete or ungrammatical responses (see Table 3 for participants’ answers to two instances of a target SVO sentence). Only 32 out of 300 responses were grammatical, 17 in the bilingual group and 15 in the monolingual group. In the monolingual group, 13/15 of the monolingual grammatical sentences were produced by one child, M5, while the other two were produced by child M4. In the bilingual group, three children produced grammatical utterances: B1 produced five grammatical utterances, B4 produced three, and B5 produced nine grammatical utterances. Only eight of all the grammatical sentences met the underlying target sentence structure (often featuring vocabulary substitutions), and seven of those eight were produced by the bilingual group: six by B5 and one by B1. The responses that included grammatical sentences produced by the children (noting the target structures) are given in Appendix A.

Due to floor effects, we could not run a statistical analysis on the participants’ number of correct answers: seven out of ten participants scored 3 or below out of a possible 90, while the remaining three participants achieved somewhat higher scores: B1 scored 18, B5 scored 13, and M5 scored 12 out of 90 correct. The number of errors was analysed instead. Although the bilinguals made fewer errors (M = 156, SD = 43.49) than the monolinguals (*M* = 165.6, SD = 23.19), an independent samples *t*-test revealed this difference not to be significant (t(8) = −0.44, *p* = 0.68), confirmed by the bootstrap confidence interval for the mean differences between the groups^3^.

To identify any finer-grained patterns, we conducted analyses that are not typically carried out in studies using the LITMUS SRep Task with neurotypical bilinguals, such as examining participants’ use of tense-marking morphemes and content vs. function words (following Polišenská et al., 2015) and qualitatively describing their errors on complex syntactic structures.

### 3.1. Content vs. Function Words

To explore whether the observed patterns were similar across groups, the percentages of CWs repeated, omitted, and substituted per group were analysed, focusing on nouns and verbs separately. Substitutions were not broken down further as there were relatively few. Independent samples *t*-tests revealed no significant differences between groups on any of these measures, confirmed by the bootstrap confidence interval for the mean differences between the groups. Table 4 reports the results on comparisons of CWs across groups together with effect sizes (Cohen’s *d*).

To analyse common error patterns in more detail, a two-way analysis of variance (ANOVA) was performed, investigating the effect of lexical category on percentage of CWs omitted, with lexical category as the within-subjects factor (two levels: nouns vs. verbs), and group as the between-subjects factor. A significant main effect of lexical category was revealed (F(1,8) = 18.3, *p* = 0.003, Ƞp^2^ = 0.696), but no significant main effect of group (F(1,8) = 0.11, *p* = 0.75, Ƞp^2^ = 0.014) and no significant interaction between group and lexical category (F(1,8) = 0.16, *p* = 0.7, Ƞp^2^ = 0.019). These results indicate that both groups omitted significantly more verbs than nouns, with no significant difference between groups.

For FWs analysis, copulas were not included, as they were only elicited in two stimuli. Not all data met the normality assumption for the independent samples *t*-test; in these cases, a Mann–Whitney test was performed. No significant differences were found between groups on any measures, again confirmed by the by the bootstrap confidence interval for the mean differences between the groups (Table 5).

To investigate whether groups differed on the percentage of CWs and FWs omitted, a two-way ANOVA was performed, with word type as the within-subjects factor (two levels: CWs vs. FWs), and group as the between-subjects factor. A statistically significant main effect of word type was revealed (F(1,8) = 53.55, *p* < 0.001, Ƞp^2^ = 0.87), but there was no significant main effect of group (F(1,8) = 0.04, *p* = 0.84, Ƞp^2^ = 0.005) and no significant interaction between group and word type (F(1,8) = 0.61, *p* = 0.46, Ƞp^2^ = 0.071), indicating that both groups omitted significantly more FWs than CWs, with no significant difference between groups.

**Figure 1:**
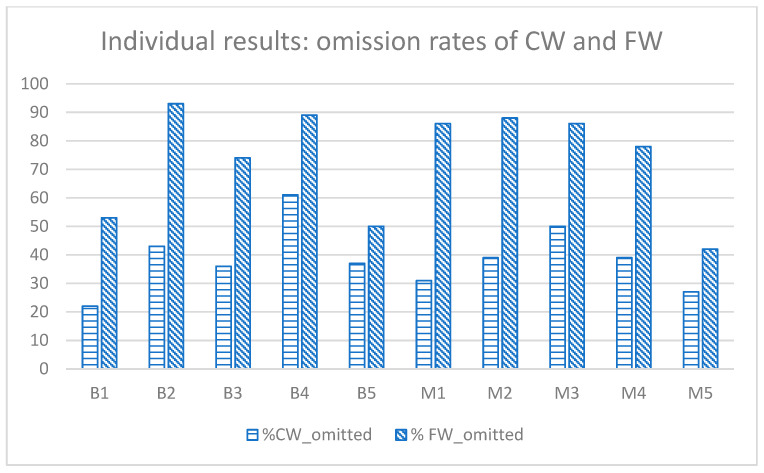
Omission rates of content words (CWs) and function words (FWs) for each child. B = Bilingual participants; M = monolingual participants.

However, given our small sample size, these analyses may obscure meaningful individual variations that are clinically relevant in populations with developmental disorders. Individual performance analyses could reveal important patterns not captured by group-level statistics. Below, we discuss individual data across relevant comparisons. Figure 1 illustrates the disparity in the omission of content versus function words. While each child omits FWs more frequently than CWs, this disparity is less pronounced for two participants in the bilingual group (B1, B5) and one participant in the monolingual group (M5).

**Figure 2:**
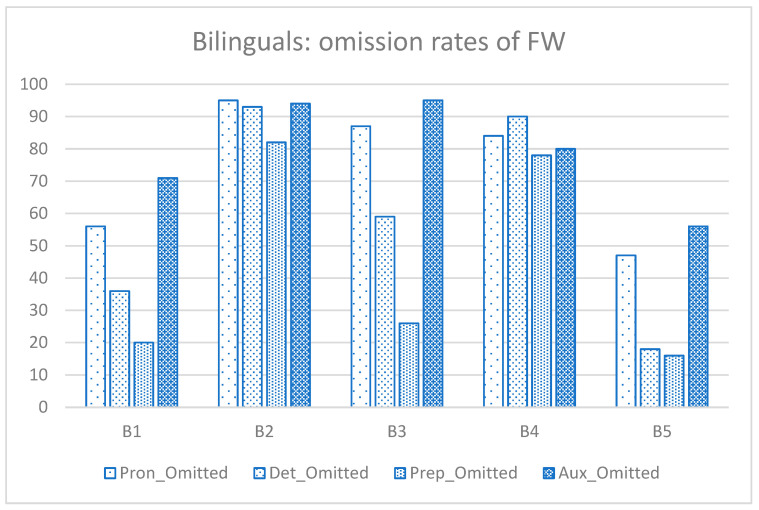
Percentage of function words (FWs) omitted by each child in the bilingual group: B = Bilingual; Pron = pronoun; Det = determiner, Prep = preposition; Aux = auxiliary.

**Figure 3:**
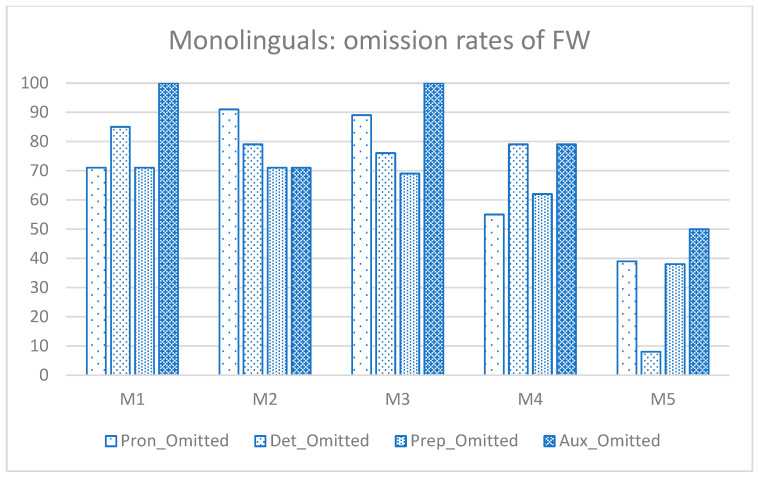
Percentage of functions words (FWs) omitted by each child in the monolingual group: M = Monolingual; Pron = pronoun; Det = determiner, Prep = preposition; Aux = auxiliary.

Figure 2 and Figure 3 present individual omission rates of FWs for each group. The bilingual group seemed to have exhibited greater within-group variability in the mastery of FWs compared with the monolingual group. As shown in Figure 2, three bilingual participants (B1, B3, and B5) had relatively low omission rates for prepositions, especially compared with pronouns and auxiliaries. B1 and B5 also performed relatively well on determiners. In contrast, Figure 3 shows that the monolingual group displayed more homogeneous performance across all FWs, with only one participant (M5) demonstrating relatively better performance on determiners, with comparable omission rates for other categories.

### 3.2. Tense

To investigate whether a pattern of more difficulty with tense-marking than non-tense-marking morphemes can be observed, we compared participants’ use of the tense morpheme ‘-ed’, vs. the non-tense morphemes progressive ‘–ing’ and plural ‘-s’. The third person singular ‘-s’ was not included as it was elicited in one stimulus only. Given the low numbers of morphemes elicited per participant, an overall percentage per group was calculated. Due to frequent CW omissions, for all participants, there were only 37 obligatory contexts for past tense ‘-ed’ (out of a possible 80), 48 for plural ‘-s’ (out of a possible 70), and 26 for ‘-ing’ (out of a possible 50). Both groups omitted the tense morpheme (-ed) more frequently than non-tense morphemes (-ing and plural -s), as observed in Table 6.

In place of the required past tense marker, children produced a non-finite form of the verb, most often an infinitive (9 out of 10), and on occasion the progressive participle. On several occasions, participants in both groups failed to produce the target FW/morpheme but did produce a correctly tensed verb (e.g., “*will feed*” → “*feeds*”; “*is patting*” → “*pats*”; “*should wash*” → “*washed*”; “*helped*” → “*was helping*”).

### 3.3. Complex Syntactic Structures: Individual Analyses

Participants produced few complex syntactic structures. Structures containing embedded clauses were often simplified and turned either into a sequence of two simple clauses (“*The child ate breakfast. He washed his face*” for the target: “The child ate breakfast after he washed his face” by participant B1) or a coordinated sentence, inserting a coordinating conjunction ‘and’ (“*A man swallowed a bee and the bee hurt him*” for the target “The bee that the man swallowed had hurt him” by participant M5). No relative clauses were produced in either group; some attempts included “*Farmer push back*” by M4 for the target: “*The horse that the farmer pushed kicked him in the back*”. One conditional was produced by M5 (“*People get a present if they clean the house*”), and one complex sentence containing an adverbial clause by B5 (“*He feeds the cow before he waters the plant*”) (see Appendix A).

To better understand our participants’ mastery of complex syntactic structure, we provide a qualitative analysis of their responses to target sentences involving object wh-questions and passives. Although statistical analysis was not feasible, the bilingual group appeared more likely to attempt these structures than the monolingual group (see Table 7 and Table 8 for all participants’ responses, with attempted target structures in bold typeface). Table 7 presents individual responses to items 17 and 25, each involving a wh-object question. One monolingual child, M4, attempted a wh-question for both items, one of which was fully grammatical (“*What did he cook?*”), compared with three bilinguals, B1, B4, and B4, who produced two fully grammatical questions (“*What did the father cook in the evening?*”; “*Who did the monkey splash near the water?*”); and one containing an omission of an aux (“*What father cook in the evening?*”). Another monolingual child, M5, converted a wh-object question into a grammatically correct declarative: “*Father was cooking in the evening*”.

Table 8 presents individual responses to items targeting two passive constructions, a short passive (without the by-phrase), item 20, and a long passive (including a by-phrase), item 21. While converting actives into passives was a common error, where for example, a short actional passive, “She was stopped at the big red lights” was converted into an active by M5 and B5 (e.g., “*She stopped at the red lights*”/“*She stopped at the signal*”), only the bilingual participants B1, B3, and B5 attempted a passive, whether short or long, two of these instances being fully grammatical: “*The mother was followed by the girl*; “*A mum was followed by a girl.*”.

## 4. Discussion

This study investigated whether bilingualism influences the expressive grammar of children with DS using the LITMUS SRep Task, an assessment specifically developed to identify language impairments in bilingual and monolingual children, targeting a range of late-developing structures (Marinis & Armon-Lotem, 2015). This is the first study to apply the LITMUS SRep Task to bilingual children with DS, extending its use beyond previously studied populations.

### 4.1. Overall Performance: Grammatical Challenges in Bilinguals and Monolinguals with DS

Our sample included children with DS, all of whom spoke English as the societal language alongside a variety of home languages and were growing up in the multilingual, multicultural context of London, UK. To account for the considerable heterogeneity in both verbal and non-verbal abilities characteristic of DS, participants were closely matched on age, receptive vocabulary and grammar, performance IQ, and the type of speech and language therapy they received. To further control for the language delays associated with this condition, we restricted the age range to 5–8 years, focusing on children who had already begun formal education within the UK system. This contrasts with previous studies that included much wider age ranges, from as young as 4 to as old as 19, potentially introducing additional developmental variability. While our quantitative results did not reveal statistically significant group differences, qualitative analyses highlighted patterns that suggest a potential bilingual advantage in specific grammatical domains. These findings contribute to the limited but growing body of research on bilingualism in DS, particularly in under-researched multilingual urban settings like London, which differ considerably from the more structured bilingual environments studied previously, such as Wales or Canada.

Our findings are in line with the broader literature documenting significant deficits in morphology and syntax among individuals with DS. Both the bilingual and monolingual groups demonstrated extremely poor performance on the LITMUS SRep Task. Seven out of ten participants scored three or fewer out of a possible 90, and the remaining three children scored no more than 18 correct. Some children repeated only two to three words per sentence, often omitting grammatical markers entirely, while others attempted more complex syntactic structures. Across 300 target sentences, only 32 utterances—just over 10%—were judged to be grammatical.

Consistent with a large body of prior research on grammatical morphology in DS (e.g., Chapman et al., 1998; Eadie et al., 2002), tense-marking morphemes and function words posed a particular challenge for all participants. High omission rates were observed across auxiliaries, modals, pronouns, determiners, and prepositions, regardless of language background. Our results also mirror earlier studies reporting verb-related difficulties in this population. Participants omitted verbs more frequently than nouns, in line with Hesketh and Chapman (1998), whose monolingual participants with DS produced fewer verbs per utterance than MLU-matched typically developing controls. A similar pattern was reported by Cleave et al. (2014), where both bilingual and monolingual children with DS showed stronger performance on nouns than verbs. Observed difficulties with producing complex sentences are consistent with previous findings in monolingual individuals with DS, who have been shown to struggle with both the comprehension and production of syntactically complex structures such as wh-questions and passive constructions (Ring & Clahsen, 2005; Joffe & Varlokosta, 2007; Perovic & Wexler, 2019). In one of the few studies to examine both constructions, and the only study that included a bespoke sentence repetition task targeting wh-questions, Joffe and Varlokosta (2007) reported that 10 monolingual children and adolescents with DS (aged 6–14) achieved only a 40% success rate in comprehending long and short passives. These same participants correctly comprehended only 50% of the wh-questions and repeated just 15% of object wh-questions.

Given that young English-speaking neurotypical children may continue to struggle with (certain types of) passives and object wh-questions even after the age of 5 (Guasti, 2017), it is unsurprising that our participants with DS, aged 5–8, were largely unable to produce these structures correctly. It is also likely that, at least for some individuals with DS, these structures may remain challenging into adulthood (see Perovic & Wexler, 2019, for persistent difficulties with different types of passives and even actives in adults with DS, potentially linked to effects of premature ageing).

### 4.2. A Bilingual Advantage?

While overall scores did not differ significantly between groups, a qualitative analysis of our participants’ error types suggests that bilingualism may confer certain advantages in specific areas of grammatical production. Of the eight sentences produced that were actual target sentences, seven were produced by the bilingual group. Moreover, the bilingual participants who produced these sentences, B1 and B5, also showed a tendency to omit fewer prepositions and determiners compared with the monolingual peers. These differences could be linked to morphological richness of the participants’ home languages: Greek and Polish, spoken by B1 and B5, are highly inflected, with complex morphosyntactic systems. Exposure to these languages may support a more robust metalinguistic awareness facilitating performance on English grammatical tasks. This is in line with findings from typical development, showing that children exposed to languages with rich inflectional morphology may show enhanced performance on certain morphosyntactic tasks (e.g., Xanthos et al., 2011). Similar effects have been observed in adults with DS: Perovic (2017) reported that adults with DS, speakers of Serbo-Croatian, another highly inflected language, showed fewer morphosyntactic errors in a narrative task compared with their English-speaking peers.

While the reported patterns are not sufficiently strong to claim a bilingual advantage (and may reflect only individual variation, common in DS), the potential influence of language typology in shaping morphosyntactic development in both typical development and DS warrants further exploration.

### 4.3. How Appropriate Is Sentence Repetition for Assessing Expressive Grammar in DS?

The poor performance of children with DS on the SRep Task here is in line with reports in the literature. Joffe and Varlokosta (2007) report 15% repetition for object wh-questions for 5–14 year olds with DS, while in Eadie et al. (2002), seven-year-olds with DS performed at about half the rate of MLU-matched typical three-year olds. Fabbro et al. (2002) report a higher percentage of correctly repeated sentences (36%) in their Italian-speaking participants with DS, compared with our 10%, though their stimuli were shorter than those in LITMUS SRep.

While sentence repetition tasks are useful for assessing the production of complex sentence structures that rarely appear in everyday contexts (e.g., passives, object wh-questions, relative clauses), they may not be suitable for young children with DS (despite being commonly included in standardised tests examining morphosyntax; cf. Ward & Sanoudaki, 2021, 2023). The extremely high error rate shown by our participants resulted in floor effects, limiting the choice of statistical analyses. Verbal working memory, known to be weak in DS, also may have played a role in our participants’ low performance. Furthermore, 7 of our 10 participants had a history of hearing loss; this could have affected their ability to accurately repeat the stimuli. Note, however, that hearing loss alone cannot fully explain the grammatical difficulties observed in our participants: two of the bilingual children who produced some of the most complex grammatical structures, B1 and B2, also had a history of hearing loss.

### 4.4. Limitations

The findings of the current study and their clinical implications should be considered in the context of the study’s limitations. Our small sample size substantially restricts the statistical power of our analyses and the generalisability of results, while qualitative analyses have limited value. Furthermore, all participants were recruited from a centre where they received weekly occupational and speech-language intervention, not typical for the UK, thus introducing selection bias. Language history data were incomplete and only obtained for four out of five bilingual participants. Bilinguals’ performance is known to be affected by the quantity and quality of input they receive (Marinis & Armon-Lotem, 2015), in addition to age of acquisition and length of exposure. No assessment in the home languages was performed, contrary to best practices in bilingual assessment (RCSLT, 2018). A comparison with bilingual typical controls would have allowed for a better understanding of whether the observed errors were caused by the combination of bilingualism with DS or just bilingualism (Paradis et al., 2011). Lastly, sentence repetition, despite its clinical value, may not be the best tool to tap into the grammar of young children with DS due to its heavy demands on verbal working memory, a domain of known weakness in this population.

## 5. Conclusions

Our results further contribute to the sparse literature on language abilities in children with DS growing up in multilingual homes in multicultural communities where the home language is a minority language that is unsupported by the educational and health system, such as the UK. In the first study to employ a sentence repetition task to compare the grammatical abilities of monolinguals and bilinguals with DS, both groups displayed similar expressive deficits on all structure types. At the same time, we uncovered suggestive findings of a bilingual advantage for complex syntactic structures, especially passives and wh-questions. While tentative, these findings call for further research in this area: on a larger population sample; with assessments administered in both languages spoken by bilingual participants; and across a wider variety of language combinations. This will help build a clearer profile of the strengths and difficulties of bilingual children with DS. Our results support the conclusions from the existing evidence on bilingualism in children with DS and, more widely, from the literature on bilingualism in children with language impairment: dual-language exposure is not detrimental to these children’s language development. The obvious clinical implications are that parents should not be discouraged from speaking to their children in more than one language. However, if the pattern revealed in our qualitative analysis is on the right track, pointing to an advantage of bilingual exposure on complex grammatical structures in the majority language, English, then parents should be encouraged to maintain their home languages.

## Figures and Tables

**Table 1:** Participants’ ages and standard scores on tests of non-verbal reasoning, receptive vocabulary, and receptive grammar.

Group	Participant ID (Language)	Age	KBIT Matrices Raw Score	KBIT Matrices SS	BPVS3 Raw Score	BPVS3 SS	TROG-2 Raw Score	TROG-2 SS
**Bilingual**	B1 (Greek)	5; 9	7	63	61	78	4	76
	B2 (Somali)	5; 9	13	88	56	75	1	62
	B3 (Swedish)	6; 5	15	90	31	<70 *	0	55
	B4 (Indonesian)	7; 4	14	79	23	<70 *	0	55
	B5 (Polish)	8; 3	12	63	60	<70 *	4	55
***Mean***		6; 8 (1.08)	12.2 (3.11)	76.6 (13.09)	46.2 (17.85)		1.8 (2.05)	60.6 (9.13)
**Monolingual**	M1	5; 4	14	96	73	95	2	74
	M2	6; 2	3	41	48	<70 *	5	74
	M3	6; 8	16	92	52	<70 *	2	55
	M4	7; 0	15	84	72	74	4	55
	M5	8; 9	11	56	82	<70 *	6	58
***Mean***		6; 9 (1.27)	11.8 (5.26)	73.8 (24.09)	65.4 (14.66)		3.8 (1.79)	63.2 (9.94)

Notes. KBIT: Kaufman Brief Intelligence Test; BPVS: British Picture Vocabulary Scales 3, TROG-2: Test of Reception of Grammar 2. SS = standard score. * BPVS3 does not offer standard scores for children with scores below 70.

**Table 2:** Examples of sentences structures in LITMUS SRep (number of items per structure).

Sentence Structure	Example
SVO with 1 auxiliary/modal (3)	They are eating the bananas in the park.
SVO with 2 aux/1 aux + 1 modal (3)	The policeman has been looking at us.
Who/What object questions (4)	What did the father cook in the evening?
Which object questions (2)	Which picture did he paint at home yesterday?
Bi-clausal sentences: complement/adjunct clauses (3)	She went to the nurse because she was sick.
Object relative clause—sentence final (3)	The children enjoyed the sweets that they tasted.
Object relative clause—centre embedded (3)	The bee that the man swallowed had hurt him.
Short passives (3)	She was stopped at the big red lights.
Long passives (3)	The mother was followed by the girl.
Conditionals (3)	If the kids behave, we will go in the garden.

**Table 3:** Participants’ responses for a target simple SVO sentence containing auxiliaries (LITMUS SRep).

**ID**	**Target: “They are eating bananas in the park” (item 1)**	
M1	bananas in the park	
M2	bananas the park	
M3	bananas	
M4	bananas in the park	
M5	eating bananas in the park	
B1	N/R	
B2	banana park	
B3	bananas in the park	
B4	nanas in the park	
B5	they are eating bananas in the volcano	
**ID**	**Target: “The policeman has been looking at us” (item 8)**	
M1	looking at us	
M2	looking us	
M3	policeman	
M4	police looking	
M5	the police was looking at us	
B1	the policeman is looking at	
B2	policeman looking us	
B3	police looking at us	
B4	looking at us	
B5	a police was looking at us	

Note: N/R = No response provided.

**Table 4:** Percentages of content words (CWs) omitted and substituted, with separate figures given for the omission of nouns and verbs.

	Bilinguals		Monolinguals		*p*-Values	Cohen’s *d*
	M	SD	M	SD		
**Total CW substituted**	8.2	5.26	9	7.07	0.84	0.13
**Total CW omitted**	36.8	14.82	34.8	8.73	0.8	0.16
**Nouns omitted**	30	13.58	28.6	10.74	0.86	0.11
**Verbs omitted**	46.6	20.96	42.4	9.5	0.69	0.26

**Table 5:** Percentages of function words (FWs) omitted and substituted.

Percentage	Bilinguals		Monolinguals		*p*-Values	Cohen’s *d*
	M	SD	M	SD		
**FWs omitted** *^a^*	69.4	21.83	75.2	18.83	0.92	−0.28
**Pronouns omitted**	73.8	22.39	69	22.27	0.74	0.22
**Determiners omitted** *^a^*	59.2	32.89	65.4	32.25	0.92	−0.19
**Prepositions omitted** *^a^*	44.4	32.72	62.2	14.03	0.6	−0.71
**Auxiliaries omitted**	79.8	18.46	78.6	22.4	0.93	0.06
**FWs substituted**	3.2	2.68	2	2	0.45	0.51

Note: *^a^* indicates that a non-parametric test was performed.

**Table 6:** Tense vs. non-tense bound morpheme omission in obligatory contexts.

Morpheme	Bilinguals		Monolinguals		Total	
	N	%	n	%	n	%
Progressive—ing	0/14	0%	0/12	0%	0/26	0%
Plural—s	3/22	13.6%	0/26	0%	3/48	6.3%
Past—ed	3/17	17.6%	7/20	35%	10/37	27.03%

**Table 7:** Participants’ responses for two object questions from LITMUS SRep: items 17 and 25.

**ID**	**Target:** “What did the father cook in the evening?” (item 17)	
M1	father cook evening	
M2	N/R	
M3	father cook	
M4	**what did he cook**	
M5	father was cooking in the evening	
B1	**what father cook in the evening**	
B2	father/toot/	
B3	cook in the evening	
B4	**what cook**	
B5	**what did the father cook in the evening**	
**ID**	**Target**: “Who did the monkey splash near the water?” (item 25)	
M1	monkey splash near water	
M2	splash […] water	
M3	monkey splash	
M4	**who he splash**	
M5	the nonkey was splashing the water	
B1	**who did the monkey splash near water**	
B2	monkey splash	
B3	splash in the water	
B4	**who splash**	
B5	**why the monkey splash big water**	

Note: Attempted wh-questions are in bold. N/R = No response provided.

**Table 8:** Participants’ responses for two passive constructions from LITMUS SRep, items 20 and 21.

**ID**	**Target:** “She was stopped at the big red lights” (item 20)	
M1	stopped big red lights	
M2	stopped on the lights	
M3	she stop lights	
M4	she stops lights	
M5	she stopped at the red lights	
B1	**she was stopped in the red lights**	
B2	big lights	
B3	stopped at big red lights	
B4	light	
B5	she stopped at the signal	
**ID**	**Target**: “The mother was followed by the girl” (item 21)	
M1	followed girls	
M2	mother … girl	
M3	mother followed girl	
M4	girl followed mum	
M5	the mother was following the children	
B1	**the mother was followed by the girl**	
B2	mummy follow	
B3	**mother followed by girls**	
B4	the girl	
B5	**a mum was followed by a girl**	

Note: Attempted passives are in bold.

## Data Availability

The data presented in this study are available on request from the corresponding author due to ethical and privacy reasons.

## Notes

1 In line with Grosjean (2010) and the Royal College of Speech and Language Therapists (RCSLT, 2018), we define bilingualism as the ability to understand and/or use more than one language, regardless of level of proficiency.

2 CWs and FWs were already identified in LITMUS SRep: for each item, the test gives the exact number of function and content words which can be used for scoring; for example, the first sentence in Table 2 above, labeled as ‘SVO with 1 auxiliary/modal’, “They are eating the bananas in the park”, the authors provide the following notation: “8 words, 3 lexical, 5 functional, 11 syllables”.

3 In addition to the parametric tests employed here, we also calculated bootstrap confidence intervals for the mean differences between the groups for each of the measures examined by independent *t*-tests. Where sample sizes are small (as they are here), bootstrap methods improve the estimates of the parameters of sampling distributions by re-sampling from the existing data set to create many samples rather than relying on any single small data set to provide estimates of means and variance. Bootstrapping has been shown to improve the accuracy of estimates without assuming that the sample or target populations are normally distributed (as parametric tests do) (see Efron & Tibshirani, 1994 and DiCiccio & Efron, 1996 for more details on bootstrapping methods). We compared the results of the *t*-tests with the conclusions drawn from the bootstrap confidence interval to check for any inconsistency. However, for each *t*-test, the bootstrap confidence interval agreed with the conclusion from the *t*-test.

## Appendix A

Grammatical sentences produced by all participants. Sentences in bold indicate sentences that met the underlying target sentence structure, albeit with some vocabulary substitutions (in italics).

**Target Structure**	**Target Sentence**	**Sentence Produced**	**Participant**
SVO + 1 aux	They are eating the bananas in the park.	**They are eating the bananas in the *volcano*.**	B5
SVO + 1 modal	The boy must sweep the floor in the kitchen.	The boy was sitting.	B5
		The boy was brushing the floor.	M5
SVO + 1 aux + 1 modal	The kitten could have hit the ball down the stairs.	The kitten hit the ball down the stairs. *	B1
SVO + 2 aux	They have been riding the goat around the garden.	They’re riding the goat around the garden.	M5
Bi-clausal sentences: complement or adjunct clauses	He will feed the cow before he waters the plants.	**He *feeds* the cow before he waters the *plant*.**	B5
	She went to the nurse because she was sick.	She went to the nurse.	B1
		She is sick.	B4
		She is sick.	M4
	The child ate breakfast after he washed his face.	The child ate breakfast/he washed his face. **	B1
Conditionals	The people will get a present if they clean the house.	**People get a present if they clean the house. *****	M5
	If the kids behave we will go in the garden.	The kids will behave in the garden.	M5
	He wouldn’t have brought his friend if she was nasty.	She is /nati/.	B4
Object relative clauses—sentence final	The mum baked the meal that the children are eating.	The mum was cooking the children.	M5
		They are eating.	B4
	He should wash the baby that the child is patting.	He washed the baby/the child is patting. ****	M5
Object relative clauses—centre embedded	The boy that the milkman helped has lost his way.	The milkman has lost his way out.	B5
		The boy was helping the man.	M5
	The bee that the man swallowed had hurt him.	A man swallowed a bee and the bee hurt him.	M5
	The horse that the farmer pushed kicked him in the back.	The farmer was kicking the horse and kicked his back.	M5
Which object questions	Which drink did the milkman spill in the house?	**Which drink did the milkman spill in the house?**	B5
What object questions	What did the father cook in the evening?	**What did the father cook in the evening?**	B5
		What did he cook?	M4
		Father was cooking in the evening.	M5
Short actional passives	The children were taken to the office.	**The children were taken to the office.**	B5
		The children went to the office.	M5
	She was stopped at the big red lights.	She stopped at the signal.	B5
		She stopped at the red lights.	M5
Long actional passives	The mother was followed by the girl.	**The mother was followed by the girl.**	B1
		***A* mum was followed by *a* girl.**	B5
		The mother was following the children.	M5
* For this sentence to be grammatical, it is assumed that ‘hit’ is used as the past tense form of the verb. However, it is also possible the child omitted the auxiliary and the modal. ** The transcription implied these were two separate utterances, both grammatical, but it is possible this was one ungrammatical sentence with the preposition omitted. *** Although the determiner was omitted, the sentence remains grammatical, though the meaning changes slightly. **** The transcription implied these were two separate utterances, and only the first one would be grammatical. However, if the child attempted to produce one complete sentence (with ‘that’ being implicit), it could be grammatical and would meet the target structure. **Total count of grammatical utterances per participant:** B1: 4 utterances; B2: none; B3: none; B4: 3 utterances; B5: 9 utterances; M1: none; M2: none; M3: none; M4: 2 utterances; M5: 13 utterances.			
